# Polygenic, cell-envelope adaptations drive high-frequency daptomycin resistance in *Staphylococcus capitis* NRCS-A from neonatal sepsis and NEC

**DOI:** 10.1128/aac.01414-25

**Published:** 2026-03-24

**Authors:** Lara Kränkel, Janna Hauser, Jessica Slavetinsky, Alina Zinser, Annika Schmidt, Mulugeta Nega, Ahmed M. A. Elsherbini, Tanja Schneider, Christian Gille, Martin Schaller, Jörg Fuchs, Andreas Peschel, Christoph Slavetinsky

**Affiliations:** 1 Interfaculty Institute of Microbiology and Infection Medicine, Cellular and Molecular Microbiology Division, University of Tübingen, Tübingen, Germany; 2 Pediatric Surgery and Urology, University Children´s Hospital Tübingen, Tübingen, Germany; 3 Microbial Genetics, Interfaculty Institute of Microbiology and Infection Medicine Tübingen (IMIT), University of Tübingen, Tübingen, Germany.; 4 Institute for Pharmaceutical Microbiology, University Hospital Bonn 39062, Bonn, Germany; 5 Department of Neonatology, University Children's Hospital Tübingen, Tübingen, Germany; 6 Department of Dermatology, University Hospital Tübingen 27203 , Tübingen, Germany; 7 Cluster of Excellence EXC 2124 Controlling Microbes to Fight Infections, Tübingen, Germany; 8 Infection Biology, Interfaculty Institute of Microbiology and Infection Medicine Tübingen (IMIT), University of Tübingen 9188, Tübingen, Germany; 9 German Center for Infection Research (DZIF), partner site Tübingen, Tübingen, Germany; The Peter Doherty Institute for Infection and Immunity, Melbourne, Victoria, Australia

**Keywords:** *S. capitis*, neonatal sepsis, LOS, NEC, NRCS-A, daptomycin resistance, bacterial cell wall

## Abstract

*Staphylococcus capitis* NRCS-A is a major cause of neonatal sepsis worldwide and exhibits resistance to multiple antibiotics. We assessed the prevalence and mechanisms of daptomycin resistance (DAP-R) in bloodstream isolates from a German neonatal intensive care unit. Ten of 11 NRCS-A isolates (91%) were resistant to daptomycin, and 18% displayed decreased susceptibility to vancomycin. Genomic analysis revealed diverse polymorphisms in genes associated with DAP-R in *Staphylococcus aureus,* but no single amino acid variant explained resistance. Phospholipid composition remained unchanged, whereas isolates displayed increased cell surface charge and cell wall thickening. Consistently, BODIPY-labeled daptomycin binding was reduced and more diffusely distributed in DAP-R NRCS-A with weaker septal enrichment. In serial passaging experiments, *S. capitis* acquired DAP-R more rapidly and robustly than *S. aureus* or *S. epidermidis* under subinhibitory concentrations of either daptomycin or vancomycin. Resequencing after *in vitro* DAP-R evolution in *S. capitis* identified newly acquired mutations in cell envelope-associated genes, including *walK* and *mprF*. These results indicate that *S. capitis* NRCS-A rapidly evolves resistance via polygenic, cell-envelope-driven mechanisms distinct from those in *S. aureus*. The high prevalence and adaptive capacity of DAP-R in neonatal isolates raise concern for therapeutic failure in neonatal intensive care.

## INTRODUCTION

The increasing incidence of bloodstream infections caused by multi-resistant bacteria presents a major global health burden, complicating the treatment of patients facing sepsis and resulting in increased mortality and morbidity rates, particularly among vulnerable populations, such as neonates (1–5). With the development of advanced neonatal intensive care and the accompanying higher survival rates of preterm-born infants, the incidence of Late-Onset Sepsis (sepsis developed >72 h after birth, LOS) has remained at least stable in the last decades, whereas the incidence of Early-Onset-Sepsis (sepsis developed <72 h after birth, EOS) decreased (6–8). Necrotizing enterocolitis (NEC) is a severe inflammatory disease of the preterm intestine that can be complicated by intestinal perforation and sepsis (9). The most frequent bacterial species causing LOS are coagulase-negative staphylococci (CoNS), such as *Staphylococcus epidermidis*, *Staphylococcus haemolyticus,* and *Staphylococcus capitis* (10, 11)*,* the latter being isolated from the bloodstream in approximately 60% of all LOS. Recently, one specific clone of *S. capitis*, designated NRCS-A by a distinctive pulse-field-gel-electrophoresis pattern, was identified as a major pathogen in the setting of neonatal intensive care units (NICUs) (12). Whole-genome sequencing (WGS) has demonstrated that NRCS-A isolates share 63 lineage-specific genes, including a conserved composite *SCCmec-SCCcad/ars/cop* cassette and genes involved in cell wall teichoic acid biosynthesis (13). The NRCS-A clone causes endemic outbreaks of neonatal LOS around the globe and is resistant to antibiotics frequently administered in NICUs, such as beta-lactams and aminoglycosides, which carries a high risk of therapy failure (12, 14, 15). Notably, the widespread administration of vancomycin, a first-line therapeutic agent for neonatal sepsis, has been proposed as a selective pressure contributing to the clonal expansion and neonatal niche specialization of NRCS-A (12). In this regard, while the NRCS-A clone often exhibits a hetero-resistant phenotype toward vancomycin, it can also acquire resistance to vancomycin significantly faster than non-NRCS-A *S. capitis* and other staphylococci species (16). Resistance to vancomycin in the NRCS-A clone is accompanied by a thickening of the cell wall and cross-resistance to daptomycin (14, 16). Daptomycin is a cyclic lipopeptide antibiotic that is used in the treatment of (neonatal) bloodstream infections with multi-resistant bacteria such as the NRCS-A clone (17–24). While its exact mode of action remains controversial, daptomycin forms stable complexes with phosphatidylglycerol (PG) headgroups and, at high concentrations, was shown to incorporate into the cell wall and induce pores in the cell membrane, thus leading to membrane depolarization and cell death (25–29). Recent advances report a direct interference with cell envelope precursors to interrupt cell wall biosynthesis (28). Hence, resistance to daptomycin in staphylococci is generally linked to changes in cell surface composition and can be acquired through mutations in genes encoding enzymes in cell wall or cell membrane biosynthesis pathways (30–33). For example, in *S. aureus*, resistance to daptomycin is frequently associated with increased cell wall thickness, as well as elevated production and translocation of the positively charged lipid lysyl-phosphatidylglycerol (Lys-PG) by the bifunctional bacterial membrane protein MprF and decreased PG content, accompanied by an overall increase in the cell surface charge (34–36). These phenotypic adaptations can be traced back to mutations in genes affecting cell membrane charge and phospholipid metabolism (*mprF, dltABCD, pgsA, cls2*), as well as regulatory and transcriptional systems controlling cell wall homeostasis (*walKR, vraSR*) or global gene expression (*rpoB, rpoC*) (37–39).

In *S. capitis*, resistance to daptomycin was associated with alterations in the two-component regulatory system *walKR*, with no detectable changes in lipid biosynthesis pathways or surface charge modulation (40). Given the paucity of data on its antibiotic resistance mechanisms and the high rate of multidrug resistance observed in *S. capitis* NRCS-A isolates, especially toward last resort antibiotics like daptomycin and vancomycin, our study aimed to (i) extend knowledge on the prevalence of DAP-R among *S. capitis* isolates from neonatal sepsis, (ii) identify genetic variants in DAP-R-associated staphylococcal genes, and (iii) assess whether *S. capitis* evolves resistance mechanisms alternative to those established in DAP-R *S. aureus*.

## MATERIALS AND METHODS

### Bacterial strains and growth conditions

*Staphylococcus capitis* and other coagulase-negative staphylococcal species (CoNS) were isolated from blood cultures at the neonatal intensive care unit (NICU) of the University Hospital of Tübingen from 2015 to 2020. This collection included 11 *S. capitis* isolates and 1 *S. epidermidis* isolate (Table S1).

### Whole-genome sequencing

Genomic DNA was extracted using the Qiagen 20/G genomic tip kit according to the manufacturer’s instructions and quantified with the Qubit double-strand DNA broad range (BR) assay kit (Thermo Fisher). Libraries were prepared using the Nextera DNA Flex Library Prep Kit (Illumina, Cat. No. 20018705) with 500 ng input DNA and indexed with IDT for Illumina DNA/RNA Unique Dual Indexes, following the manufacturer’s protocol. Library fragment size and quality were assessed using the Agilent 2100 Bioanalyzer using the High Sensitivity DNA Analysis Kit, and concentrations were confirmed using the Qubit dsDNA High Sensitivity Assay Kit (Thermo Fisher Scientific). Equimolar pooled libraries were sequenced on an Illumina NovaSeq 6000 platform using the NovaSeq 5000/6000 S1 Reagent Kit (300 cycles) in paired-end mode (2 × 150 bp). Respective sequences were deposited in NCBI under BioProject PRJNA1330851. Whole-genome sequences of *S. capitis* NRCS-A and the reference strain *S. capitis* DSM6717 were compared to identify single-nucleotide polymorphisms (SNPs) in genes associated with cell wall biosynthesis. Annotated genomes were screened for cell wall-related genes, and sequence alignments were performed using SeqBuilder (DNASTAR). The reference strain *Staphylococcus capitis* DSM6717, originally isolated from human skin, was obtained from the DSMZ culture collection (Braunschweig, Germany). In addition to our CoNS strain collection, we included well-characterized *Staphylococcus aureus* strains: the methicillin-susceptible laboratory strain *S. aureus* SA113, the methicillin-resistant clinical clone *S. aureus* USA300, and the SA113 *mprF* knockout derivative SA113 *ΔmprF*, which has been described previously (41). Strains were routinely cultured in Tryptic soy broth (TSB) and, for antibiotic susceptibility testing, in Mueller–Hinton broth (MHB). Cultures were incubated at 37°C on an orbital shaker at 160 rpm for strain maintenance or respective analysis.

### Antibiotic resistance and DAP-R evolution analysis

To detect DAP-R and/or vancomycin-resistant (VAN-R) *S. capitis* isolates, minimal inhibitory concentrations (MICs) were determined. Therefore, MIC test strips for daptomycin and vancomycin (Liofilchem) were applied on MHB agar plates and analyzed after 24 h incubation at 37°C according to the manufacturer’s protocol.

For DAP-R evolution experiments, strains were grown in MHB in a 48-well-plate under variable concentrations of daptomycin (starting with 0.25, 0.5, 1, 2, and 4 µg/mL) and 50 µg/mL calcium, without antibiotics or under variable concentrations of vancomycin (starting with 0.5, 1, 2, 4, and 8 µg/mL). For serial passaging, cultures from the highest antibiotic concentration that still permitted growth were used for the following day, respectively. If resistance was acquired, the highest concentration of the antibiotic was doubled to always allow subinhibitory and inhibitory concentrations for both daptomycin and vancomycin. After 24 h of incubation in an orbital shaker, the growth was measured at optical density (OD)_600_. To determine a potential change in daptomycin susceptibility, the MIC was determined after each passage via MIC test strips as described above. After 10 days of daptomycin or vancomycin passage or without antibiotic pressure, genomic DNA was isolated from respective *S. capitis* strains for WGS to identify newly evolved SNPs as described above.

Daptomycin binding to *S. capitis* isolates was assessed by flow cytometry using BODIPY-labeled daptomycin. BODIPY 493/503 (4,4-Difluoro-1,3,5,7,8-Pentamethyl-4-Bora-3a,4a-Diaza-s-Indacen) (Invitrogen/Thermo-Fisher, D3922) labeling of daptomycin was performed as described previously (42, 43). Prior to analysis, the MIC of BODIPY-labeled daptomycin was determined by microbroth dilutions using a starting OD₆₀₀ of 0.05 (Fig. S1). For flow cytometry analysis, bacteria were grown overnight in TSB supplemented with CaCl₂ (1.25 mM), diluted into fresh medium, and incubated for 2 h. Cultures were adjusted to an OD₆₀₀ of 0.15 and incubated with BODIPY-labeled daptomycin at subinhibitory concentrations (7.81 µg/mL) in the presence of CaCl₂. Samples were collected after 0, 30, 60, and 120 min, washed once with phosphate-buffered saline (PBS), and analyzed on a BD LSRFortessa flow cytometer. Daptomycin binding was quantified by measuring BODIPY fluorescence in the FITC channel. To visualize the binding of daptomycin to the bacterial cell wall, fluorescence microscopy was performed with ScSK1 and DSM6717. Bacteria were grown overnight in TSB supplemented with CaCl₂ (1.25 mM) and, the next day, to the exponential phase in the same medium. For BODIPY labeling, bacteria were washed twice with PBS, fixed with 3.3% paraformaldehyde for 15 min, and washed again with PBS. BODIPY-labeled bacteria were embedded in 1% agarose. Fluorescence was measured using a Zeiss LSM 800 microscope at 100× magnification.

### Determination of the bacterial cell surface charge

To determine the cell surface charge of *S. capitis* strains, a cytochrome c (CytC) binding assay was performed as described previously (44). Briefly, *S. capitis* strains were grown to exponential phase (OD_600_ 1), harvested, and washed twice with MOPS (3-(N-morpholino) propanesulfonic acid) (20 mM, pH 7). Afterward, cells were adjusted to OD600 3, pelleted, resuspended in 750 μL CytC solution (Sigma; 0.25 mg/mL in MOPS buffer), and incubated at 37°C for 15 min. Cell suspensions were centrifuged to pelletize cells, and the resulting supernatant was taken out and diluted 1:5 with MOPS buffer. Unbound CytC was photometrically measured at 410 nm.

### Cell wall analysis

Transmission electron microscopy (TEM) was performed as described recently (45). In brief, *S. capitis* strains were grown to stationary phase and fixed at an OD600 of 10 in 200 μL Karnovsky’s fixative (3% formaldehyde, 2.5% glutaraldehyde in 0.1 M phosphate buffer pH 7.4) for 24 h. After centrifugation at 1,400 rcf for 5 min, the supernatant was discarded, and pellets were resuspended in 20 μL agarose (3.9%) at 37°C, cooled to room temperature, and cut into small pieces. Post-fixation was carried out in 1% osmium tetroxide containing 2.5% potassium ferrocyanide (Morphisto) for 2 h. Samples were embedded in glycide ether and cut using an ultramicrotome (Ultracut E, Reichert). Ultra-thin sections (30 nm) were mounted on copper grids and analyzed using a Zeiss LIBRA 120 transmission electron microscope (Carl Zeiss) operating at 120 kV.

### Isolation and quantification of polar lipids

*S. capitis* cultures were grown to the exponential phase (OD_600_ of 1). Polar lipids were extracted using the Bligh–Dyer method (46). In brief, cells were resuspended with chloroform/methanol/sodium acetate buffer (20 mM) (1:1:1, by vol), vacuum-dried, and resuspended in chloroform/methanol (2:1, by vol). Extracts were filled into a 100 µL Hamilton syringe and spotted onto silica gel 60 F254 high-performance thin-layer chromatography plates (Merck) with a Linomat five sample application unit (Camag) and run in a developing chamber ADC 2 (Camag) with a running solution composed of chloroform-methanol-water (65:25:4, by vol) and in one dimension. Phosphate group-containing lipids were detected by molybdenum blue staining, and phospholipids were quantified in relation to total phospholipid content by determining lipid spot intensities densitometrically with ImageJ (https://imagej.net/ij/docs/guide/index.html) as described previously (47). Isolations of polar lipids from *S. aureus* SA113 and lysyl phosphatidylglycerol-deficient *mprF* mutant served as controls. A representative TLC plate is shown in Fig. S2.

### Isolation of WTA and determination of D-Ala content

WTA isolation was carried out as described previously (48), with minor modifications. Briefly, bacterial strains were grown in 50 mL of TSB supplemented with 0.2% glucose for 4 h to the exponential phase taken from overnight cultures in TSB. Cells were harvested, washed, and resuspended in 20 mM ammonium acetate (AA) buffer. Bacteria were mechanically disrupted with glass beads by agitation via FastPrep (50% cells in AA, 50% glass beads). Lysis was achieved by 5 cycles of agitation, each for 1.5 min at 14,000 rcf, and samples were cooled on ice between each cycle. The resulting cell lysate was treated with 2,540 U/mL DNAse and 115.5 U/mL RNAse in the presence of 5 mM MgCl_2_ for 5 h at 37°C. For the removal of proteins, 2% SDS was added and incubated for 1 h at 65°C. To remove residual SDS, samples were washed 11 times with 1 mL of AA. To separate the WTA from other cell wall components, samples were treated with 5% TCA (trichloroacetic acid) for 4 h at 65°C. Peptidoglycan debris was removed via centrifugation (10 min, 14,500 rcf). The purified WTA was quantified via phosphate content as previously described (49). WTA was further hydrolyzed with 100 mM NaOH at 60°C for 2 h. D-Alanine content of the WTA was determined by high-performance liquid chromatography (HPLC) using pre-column derivatization of the amino acid by ortho-phthalaldehyde (OPA). Sample and reagent (OPA diluted 1:10 in 1 M sodium borate buffer pH 10.7) [PA1] were drawn into the autosampler injection needle (Agilent 1200 HPLC system, Waldbronn, Germany) and shaken directly in the needle for 90 s according to an injector program [PA2] [CS3] before injection. The amino acid derivatives were separated on a reversed-phase column (Grom-Sil OPA-1, 150 mm × 4.6 mm, 3 µm, Alltech-Grom GmbH, Rottenburg-Hailfingen, Germany) at a flow rate of 1.1 mL/min using a linear gradient elution from 0 to 60% buffer B in 15 min and detected at 340 nm. Buffer A was 25 mM phosphate buffer (pH 7.2) containing 0.75% tetrahydrofuran (THF), while buffer B was composed of 35% MeOH and 15% acetonitrile (ACN) in 25 mM phosphate buffer. A minimum of three independent runs were performed. Peak areas were analyzed with the chemstation software, and quantification was made based on a D-alanine standard curve.

### Statistics

Statistical analyses were performed with GraphPad Prism version 10.0.0 (GraphPad Software). Group differences were analyzed with one-way or two-way analysis of variance (ANOVA) and the *P* value of ≤0.05 was considered statistically significant.

## RESULTS

### *S. capitis* isolates from neonatal sepsis reveal high rates of daptomycin resistance

**Fig 1:**
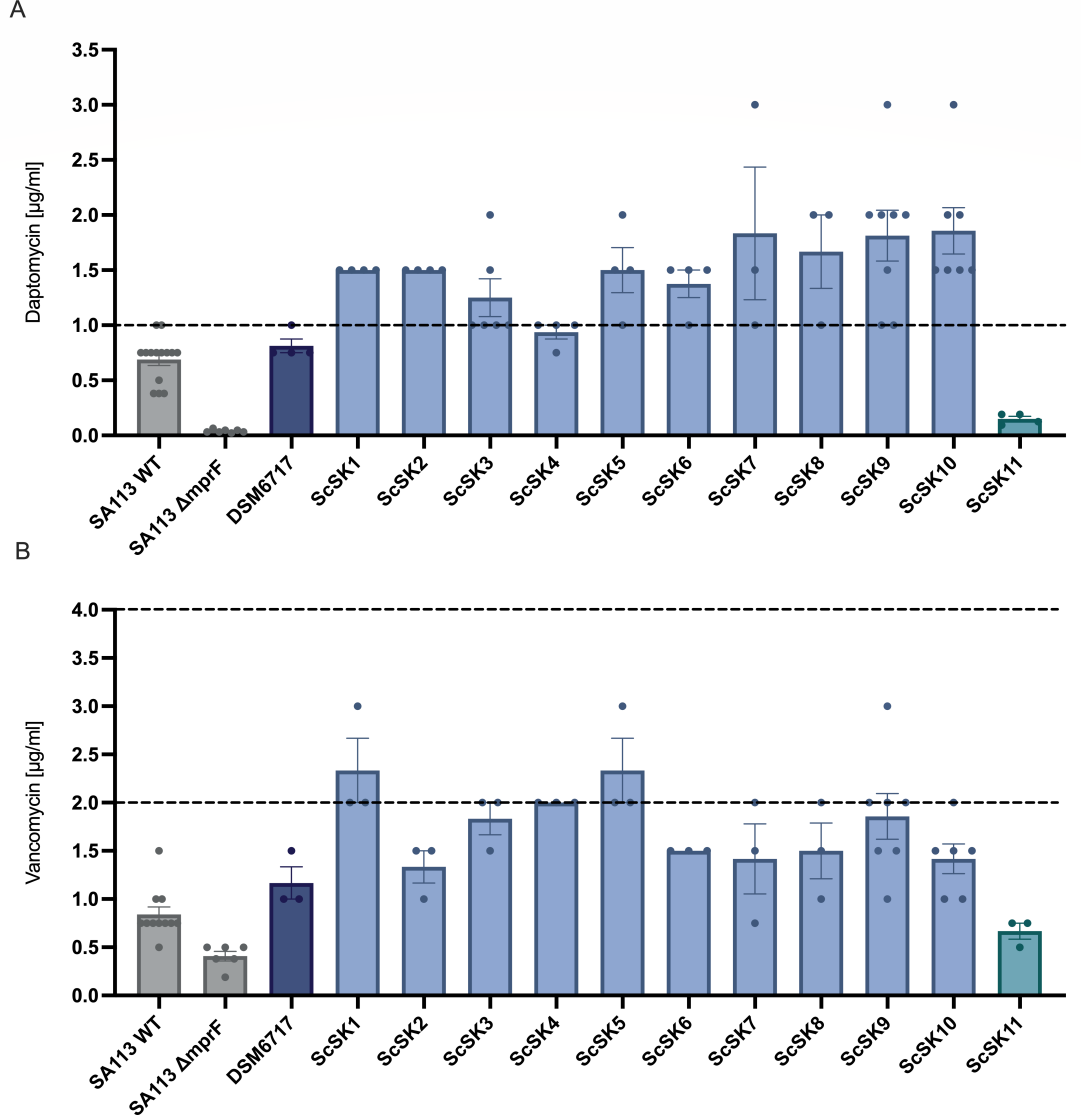
Daptomycin and vancomycin susceptibility of *S. capitis* NRCS-A isolates (ScSK) from our local collection as determined by MIC test strip. MICs of (**A**) daptomycin and (**B**) vancomycin are shown. Black lines indicate cut-off concentrations for resistance: 1 μg/mL for daptomycin, 2 μg/mL for vancomycin reduced susceptibility (cut-off for *S. aureus*), and 4 μg/mL for vancomycin resistance (EUCAST definition, v15.0 of 2025). The *S. aureus* SA113 WT and SA113 ∆*mprF* (gray), which is more susceptible to daptomycin and vancomycin, were used as controls. NRCS-A isolates (light blue) were compared with non-invasive *S. capitis* strain DSM6717 (dark blue) and the *S. capitis* bloodstream isolate from a toddler’s catheter sepsis (ScSK11, turquoise). The mean ± SEM of at least three independent experiments is shown.

**Fig 2:**
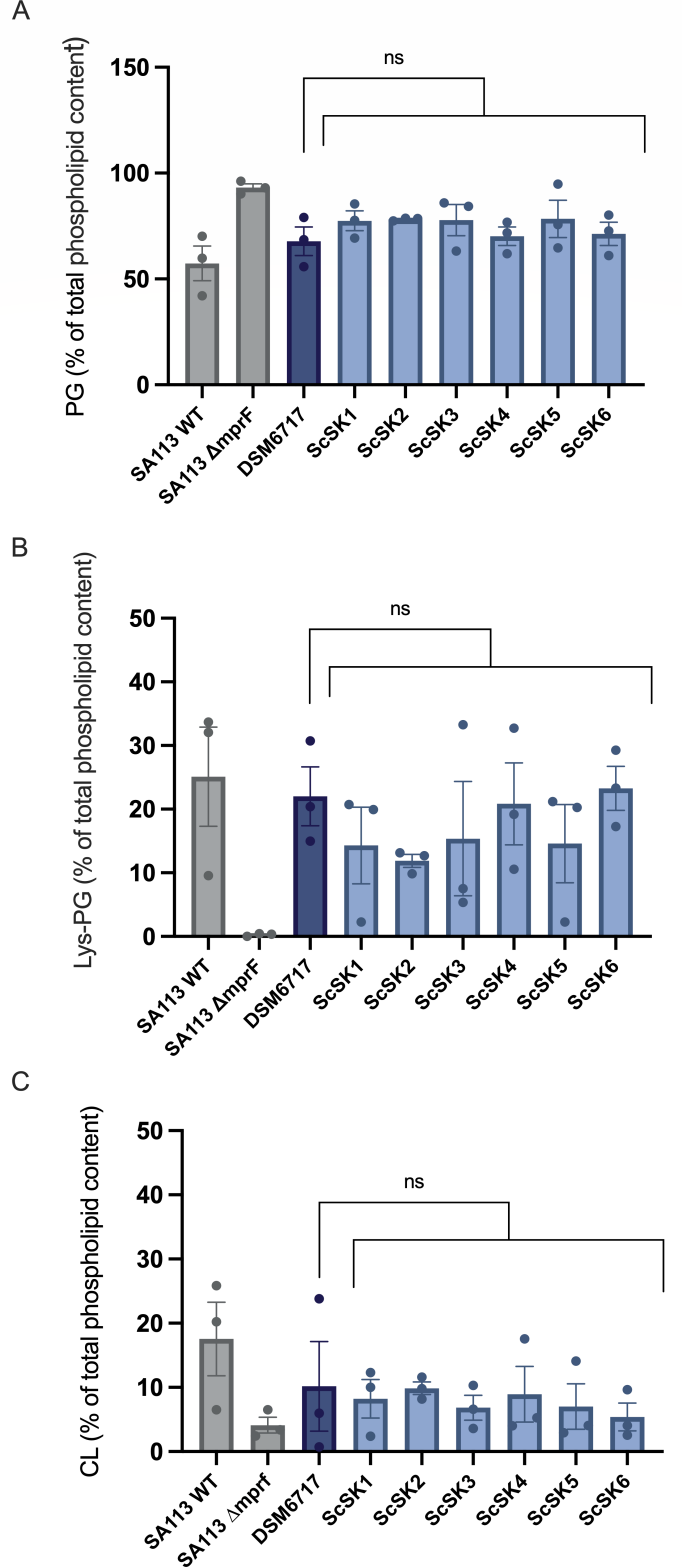
Polar lipid content of *S. capitis* NRCS-A isolates (ScSK) and non-invasive *S. capitis* strain DSM6717. Polar lipids were separated by TLC, stained with the phosphate group-specific dye molybdenum blue, and quantified densitometrically. *S. aureus* strains SA113 WT and SA113 ∆*mprf* (no Lys-PG production) were used as control strains. (**A**) Quantification of phosphatidylglycerol (PG) per indicated strain. (**B**) Quantification of lysyl-phosphatidylglycerol (Lys-PG) and (**C**) cardiolipin (CL) content per indicated strain. The mean ± SEM of three independent experiments is shown. Group differences were analyzed with one-way ANOVA with Dunnett’s multiple comparisons.

To investigate the mechanisms of multidrug resistance in *S. capitis* NRCS-A, we isolated strains from blood cultures of clinically evident neonatal sepsis in our neonatal intensive care unit (NICU) at the University Hospital of Tuebingen, Germany, between 2015 and 2020. Isolates were then assigned to the NRCS-A lineage (14) by phylogeny after whole-genome sequencing. Our NICU collection comprised 11 *S. capitis* NRCS-A from neonatal sepsis, 3 of which derived from 2 patients presenting as septic necrotizing enterocolitis (ScSK2, ScSK9, and ScKS10) with 2 genetically identical isolates (ScSK9 and ScKS10) detected in both blood culture and intraoperative swab after intestinal perforation from 1 single patient (Table S1). Prior to bloodstream infection with NRCS-A, most patients received (pre-emptive) ampicillin and tobramycin treatment; none received daptomycin, and one received vancomycin (Table S2). To subsequently analyze the mechanisms of DAP-R in our strain collection, we first evaluated the minimal inhibitory concentrations (MIC) for daptomycin and vancomycin due to their frequent cross-resistance. As a quality check, the *S. aureus* SA113 wildtype and *mprF* deletion mutant (known increased susceptibility) showed previously reported MICs for both antibiotics (50), while the *S. capitis* skin isolate DSM6717 and the bloodstream isolate from a toddler’s catheter sepsis ScSK11 (no neonatal sepsis) served as controls and showed no increased MIC for daptomycin or vancomycin (Fig. 1A and B). We found 10 of 11 (90.9%) NRCS-A isolates to be resistant to daptomycin (MIC > 1 µg/mL; EUCAST definition, v15.0 of 2025) (Fig. 1A) and 2 of 11 (18.2%) to exhibit a reduced susceptibility to vancomycin (MIC > 2 µg/mL; EUCAST definition, v15.0 of 2025) (Fig. 1B). Of note, the vancomycin MICs of most NRCS-A isolates were elevated compared to control strains (Fig. 1B). Thus, NRCS-A isolates from neonatal sepsis show high rates of daptomycin resistance and an elevated MIC for vancomycin.

### *S. capitis* NRCS-A isolates from neonatal sepsis harbor multiple genetic variations in genes associated with DAP-R in *S. aureus*

Various genetic variants have been associated with DAP-R in *S. aureus* (e.g., *mprF*, *dltABCD*, *cls2*, *pgsA*, *rpoB*, *rpoC*, *vraR*, *vraS*, *walK*, *walR*) (51–53). To explore genetic variants in these genes in *S. capitis*, we analyzed the respective sequences in the genomes of the NRCS-A collection (Table 1). Compared to the genome of the daptomycin-sensitive *S. capitis* DSM6717, genetic variants were found in *mprF*, *dltA*, *dltB*, *dltD*, *cls2*, *pgsA*, *rpoB*, *vraS*, and *walk,* but not in *dltC*, *rpoC*, *vraR*, or *walR* (Table 1). However, the daptomycin-sensitive (DAP-S) isolate ScSK4 exhibited the same genetic variations as the DAP-R isolates, with the exception of the amino acid (aa) variant N502 in *walK*. At this residue, ScSK4 showed a threonine (T502) (Table 1). Since the DAP-R isolate ScSK1 and the control strain DSM6717 exhibited the T502 variant, no single mutation could be associated with the DAP-R phenotype of our *S. capitis* NRCS-A isolates. Nevertheless, a combination of genetic variants could lead to phenotypic changes that give the decisive impetus to transition to resistance.

**TABLE 1:** SNPs found in cell wall-related genes (mprF, dltA, dltB, dltD, Cls2, pgsA, rboB, vraS, walK) in *S. capitis* NRCS-A (ScSK) isolates^*a*^

Strain	Dap-R/S	mprF	dltA	dltB	dltD	Cls2	pgsA	rboB	vraS	walK
ScSK1	R	**F336**	**D17; P227; R432; I439; K465**	**W161; L237**	**R119**	**G22**	**E187**	**T382**	**V89**	T502
ScSK2	R	**F336**	**D17; P227; R432; I439; K465**	**W161; L237**	**R119**	**G22**	**E187**	**T382**	**V89**	**N502**
ScSK3	R	**F336**	**D17; P227; R432; I439; K465**	**W161; L237**	**R119**	**G22**	**E187**	**T382**	**V89**	**N502**
ScSK4	S	**F336**	**D17; P227; R432; I439; K465**	**W161; L237**	**R119**	**G22**	**E187**	**T382**	**V89**	T502
ScSK5	R	**F336**	**D17; P227; R432; I439; K465**	**W161; L237**	**R119**	**G22**	**E187**	**T382**	**V89**	**N502**
ScSK6	R	**F336**	**D17; P227; R432; I439; K465**	**W161; L237**	**R119**	**G22**	**E187**	**T382**	**V89**	**N502**
ScSK7	R	**F336**	**D17; P227; R432; I439; K465**	**W161; L237**	**R119**	**G22**	**E187**	**T382**	**V89**	**N502**
ScSK8	R	**F336**	**D17; P227; R432; I439; K465**	**W161; L237**	**R119**	**G22**	**E187**	**T382**	**V89**	**N502**
ScSK9	R	**F336**	**D17; P227; R432; I439; K465**	**W161; L237**	**R119**	**G22**	**E187**	**T382**	**V89**	**N502**
ScSK10	R	**F336**	**D17; P227; R432; I439; K465**	**W161; L237**	**R119**	**G22**	**E187**	**T382**	**V89**	**N502**
DSM6717	S	L336	E17; S227; K432; M439; Q465	C161; F237	G119	D22	D187	A382	E89	T502

^*a*^ Bold font indicates variations in the genomes of ScSK strains compared to the genome of *S. capitis* DSM6717. no SNPs were found in dltC, vraR, rboC, and walR (data not shown).

### *S. capitis* NRCS-A isolates show no altered phospholipid content

Since DAP-R in *S. aureus* is associated with changes in the levels of PG, cardiolipin (CL), and Lys-PG (39, 54–56), we speculated that similar membrane lipid alterations could be present in our *S. capitis* NRCS-A isolates that carried mutations in the corresponding biosynthesis genes (*pgsA*, *cls2*, and *mprF*, respectively) (Table 1). Therefore, we analyzed the phospholipid content of six representative NRCS-A isolates, including the DAP-S ScSK4. Compared to *S. capitis* DSM6717, as well as *S. aureus* SA113 wildtype, we found no significant differences in the content of PG, CL, or Lys-PG in the representative NRCS-A isolates (Fig. 2A through C), thus ruling out daptomycin resistance due to changes in phospholipid content.

### *S. capitis* NRCS-A exhibits increased cell surface charge and cell wall thickness with reduced daptomycin binding

**Fig 3:**
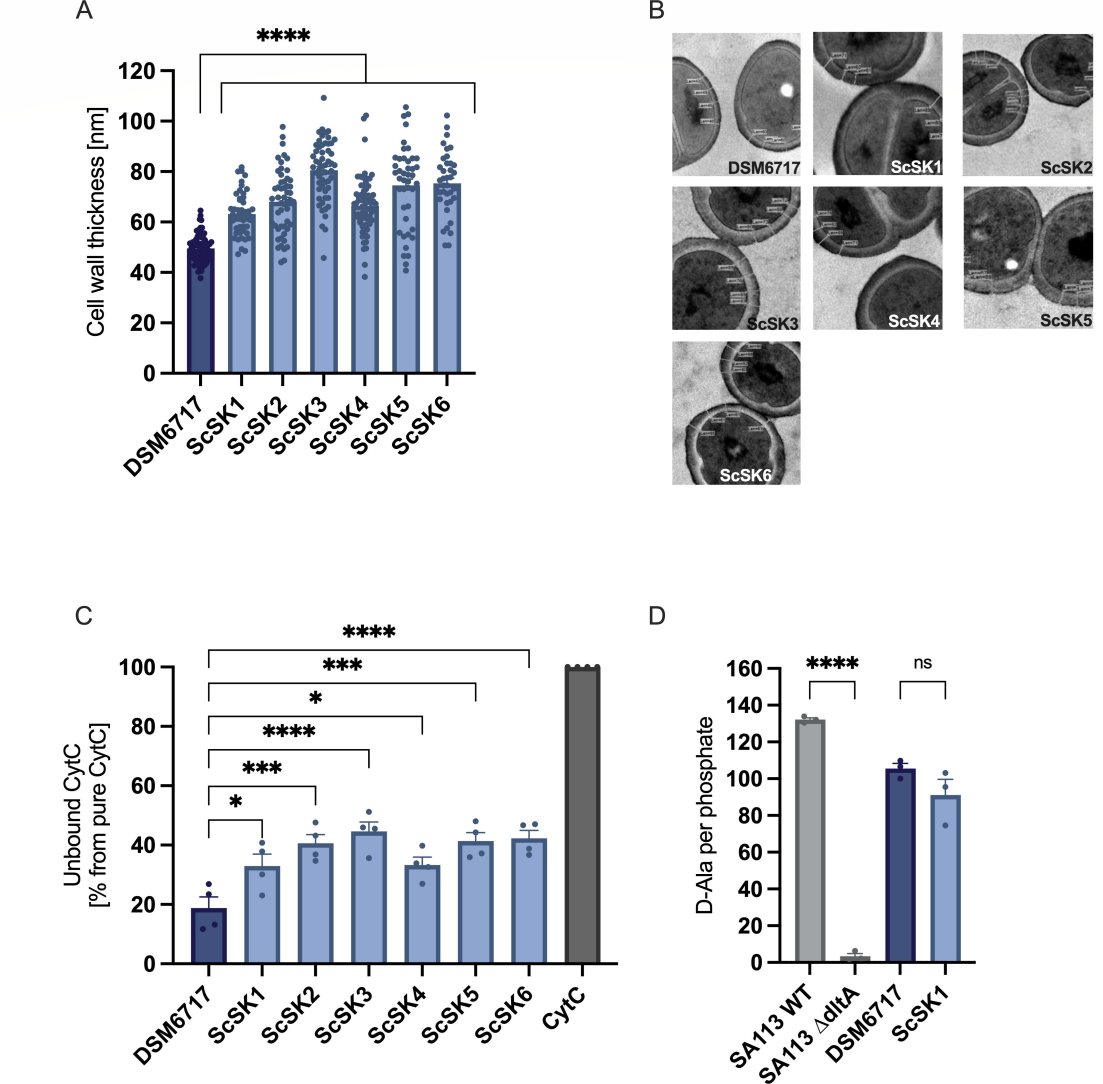
Cell wall thickness, cell surface charge, and D-alanine (D-Ala) content of *S. capitis* NRCS-A isolates from our local cohort (ScSK). (**A**) Cell wall thickness of NRCS-A isolates (light blue) compared to non-invasive *S. capitis* DSM6717 (dark blue) as determined by TEM. At least four individual measurements were performed per single cell, measuring at least 40 single cells per isolate. (**B**) Exemplary TEM pictures of respective strains. (**C**) Cell surface charge of NRCS-A isolates and non-invasive *S. capitis* strain DSM6717 was measured by cytochrome c binding assay. Pure cytochrome c solution was normalized to 100%. (**D**) D-alanine content of WTA isolated from NRCS-A strains in comparison to *S. capitis* DSM6717 as determined by HPLC. *S. aureus* strains SA113 WT and SA113 ∆*dltA* (gray) were used as positive and negative control, respectively. Group differences were analyzed with one-way ANOVA with Dunnett’s multiple comparisons (*, *P* < 0.05; ***; *P* < 0.001; ****; *P* < 0.0001). The mean ± SEM of at least three independent experiments is shown.

**Fig 4:**
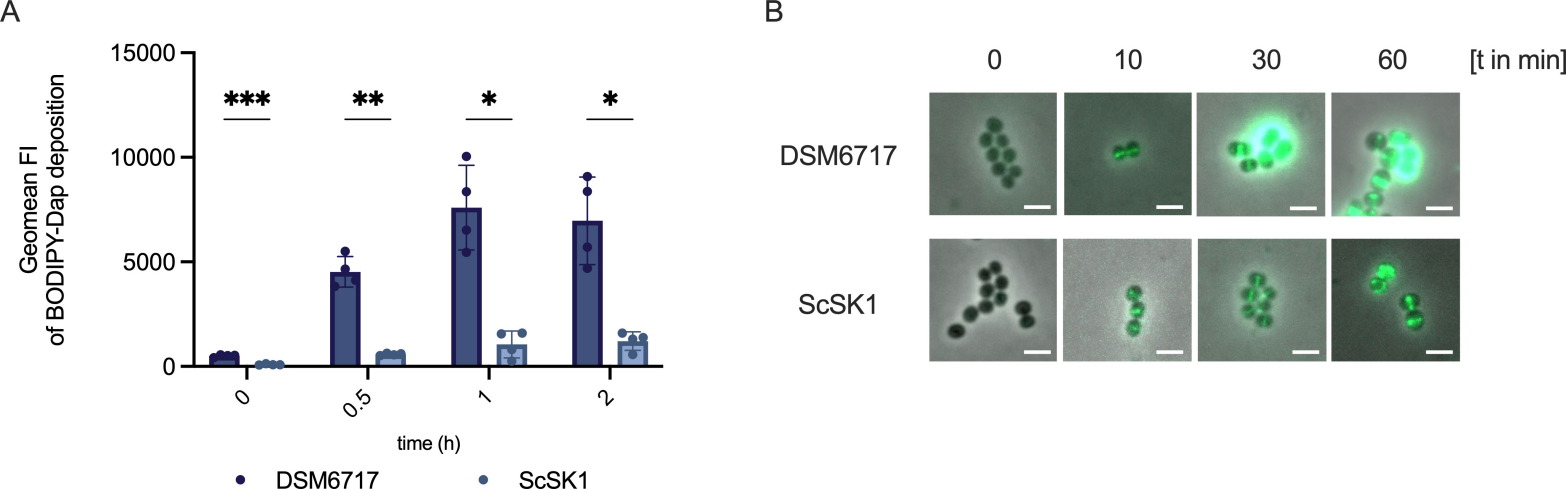
(**A**) Binding of BODIPY-labeled daptomycin (BODIPY-Dap) in the previously determined subinhibitory concentration of 7.81 µg/mL (Fig. S1) to the *S. capitis* NRCS-A isolate ScSK1 (light blue) and the non-invasive *S. capitis* DSM6717 (dark blue). Bacterial cells were incubated with BODIPY-Dap and analyzed by flow cytometry (FITC channel) at 0 h, 0.5 h, 1 h, and 2 h. Statistical analysis was performed using two-way ANOVA with Šidák’s multiple-comparisons test to compare BODIPY-Dap binding between strains at each time point (*P* < 0.05, *P < 0.01, **P < 0.001). Data are shown as mean ± SD from at least four independent experiments. (**B**) Fluorescence microscopy of *S. capitis* strains DSM6717 and ScSK1 after incubation with BODIPY-Dap for 0, 10, 30, and 60 min. Representative images were acquired on a Zeiss LSM 800 confocal microscope using a 100× objective. Scale bar, 2 µm.

An increased cell surface charge (linked to variants in *mprF* or *dltABCD*) or cell wall thickening (linked to variants in *walKR*, *vraSR, or rpoB/C*) is frequently associated with DAP-R or decreased vancomycin susceptibility in *S. aureus* (33, 50, 51, 57, 58). We found that compared to the reported cell wall thickness of *S. aureus* (20–30 nm) (59), *S. capitis,* including the control isolate DSM6717 (37.75–62.5 nm), displayed a thicker cell wall (37.75–90 nm) (Fig. 3A and B). Interestingly, all six representative NRCS-A isolates analyzed, including DAP-S ScSK4, showed a significantly thicker cell wall compared to DSM6717 (Fig. 3A and B). A cytochrome C (CytC) repulsion assay was performed to evaluate the cell surface charge of the six representative NRCS-A isolates. In this assay, the levels of unbound CytC in the supernatant after incubation of this highly cationic protein with bacteria serve as a proxy for a more positive cell surface charge. All NRCS-A isolates displayed increased levels of unbound CytC compared to DSM6717 (Fig. 3C). This result was unexpected since we found no alterations in the phospholipid content of our NRCS-A isolates. As an increase in D-alanylation of wall teichoic acids can also enhance cell surface charge in staphylococci (49), we analyzed the D-alanine content of wall teichoic acids in the DAP-R NRCS-A isolate ScSK1. Compared to *S. capitis* DSM6717, as well as *S. aureus* SA113 wildtype and *dltA* deletion mutant (lacking D-alanylation of wall teichoic acids), no significant difference in wall teichoic acid D-alanylation was observed in ScSK1 (Fig. 3D). In DAP-R *S. aureus*, daptomycin binding and envelope deposition are typically reduced, often accompanied by weaker septal enrichment (28). Therefore, we analyzed binding of BODIPY-labeled daptomycin (BODIPY-Dap) to the DAP-R NRCS-A isolate ScSK1 in comparison with *S. capitis* DSM6717. Upon incubation with BODIPY-Dap, ScSK1 displayed significantly reduced BODIPY-Dap deposition at 30 min, 1 h, and 2 h post-incubation (Fig. 4A). Consistently, fluorescence microscopy after 30- and 60-min exposure revealed numerous uniformly bright cells in the non-invasive DAP-S DSM6717, whereas the DAP-R isolate ScSK1 exhibited lower overall fluorescence intensity. In ScSK1, BODIPY-Dap staining was less enriched at the division septum and instead appeared as a weaker, more diffuse and patchier signal (e.g., at 60 min) (Fig. 4B). Thus, NRCS-A isolates from neonatal sepsis exhibit an increased cell surface charge and cell wall thickness, irrespective of daptomycin susceptibility. However, BODIPY-Dap analyses indicate that DAP-R NRCS-A isolates display reduced and more diffusely distributed envelope deposition of daptomycin; the mechanistic basis of these envelope changes remains unclear.

### *S. capitis* evolves DAP-R under daptomycin or vancomycin pressure more rapidly and robustly than *S. epidermidis* and *S. aureus* and acquires mutations in cell envelope genes

**Fig 5:**
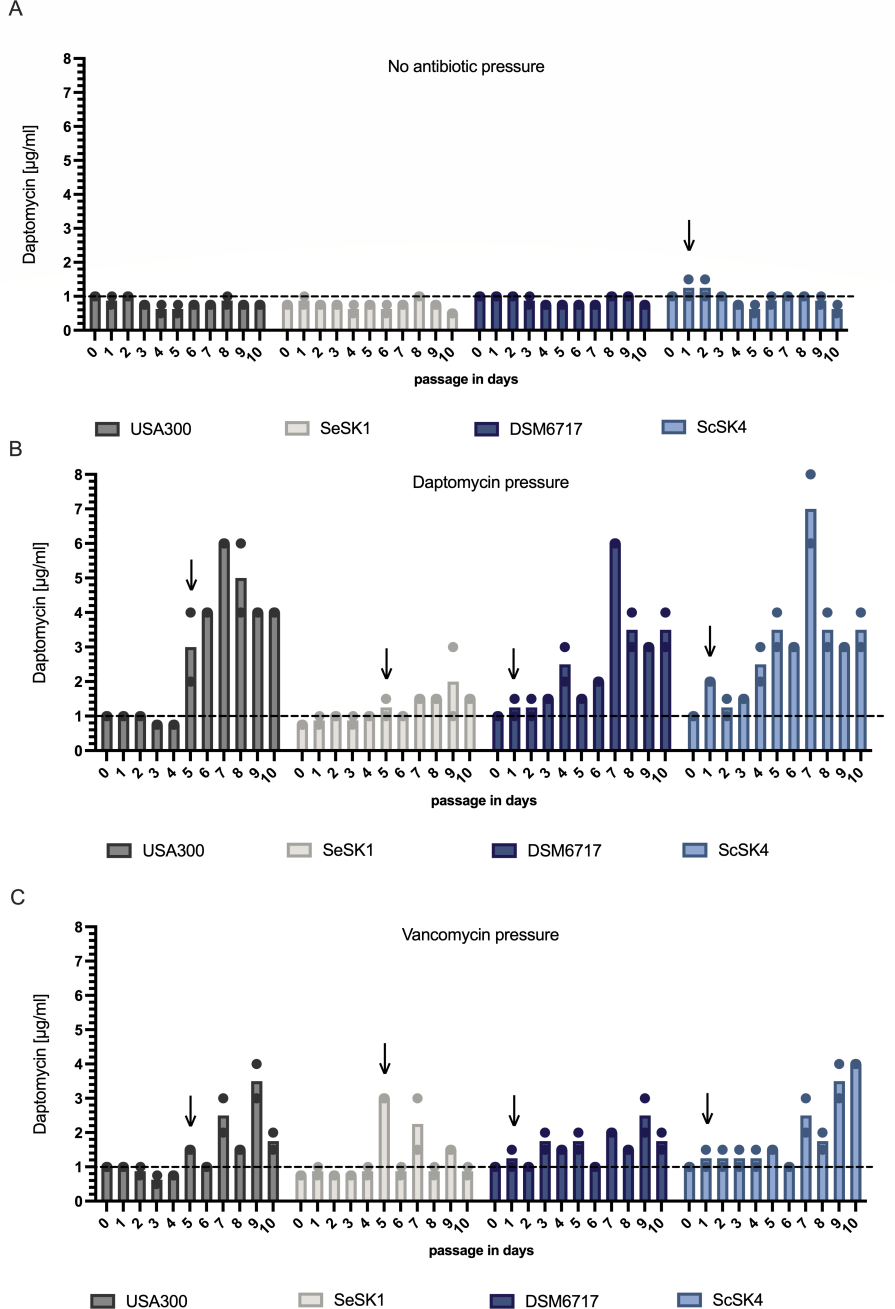
DAP-R evolution of *S. capitis* NRCS-A (ScSK4) in light blue compared to non-invasive *S. capitis* (DSM6717) in dark blue, *S. aureus* (USA300) in gray, and *S. epidermidis* (SeSK1) in white. Strains were grown without (**A**) or with (**B**) daptomycin pressure or (**C**) vancomycin pressure in MHB for 24 h followed by daptomycin MIC determination after each passage via E-tests. Black lines indicate cut-off concentrations for daptomycin resistance at 1 μg/mL (EUCAST definition, v15.0 of 2025). The black arrows highlight the passaging days on which bacterial strains evolved a DAP-R phenotype.

We hypothesized that the intrinsic resistance profile of *S. capitis* (e.g., increased cell wall thickness relative to *S. aureus*), together with the accumulation of specific genetic mutations in DAP-R-associated genes (Table 1), could be sufficient to facilitate the transition to DAP-R in *S. capitis* NRCS-A isolates. Therefore, we conducted passaging experiments over 10 days in liquid culture with (i) subinhibitory daptomycin concentrations, (ii) subinhibitory vancomycin concentrations, or (iii) without antibiotic pressure, comparing the ability of the DAP-S *S. capitis* NRCS-A isolate ScSK4, the DAP-S *S. capitis* DSM6717, the DAP-S *S. aureus* MRSA strain USA300, and the DAP-S *S. epidermidis* isolate SeSK1 from neonatal sepsis to transition to daptomycin resistance. MICs for daptomycin were determined every 24 h. In the absence of antibiotic pressure, the NRCS-A isolate ScSK4 was the only strain exceeding the MIC of 1 µg/mL for daptomycin and showed transient DAP-R on passage days 1 and 2, followed by a spontaneous normalization of its daptomycin MIC below 1 µg/mL for the remaining passages (Fig. 5A). Under antibiotic pressure with either daptomycin or vancomycin, both the *S. capitis* DSM6717 and ScSK4 rapidly evolved DAP-R on passage day 1, with increasing daptomycin MICs for the remaining passages (Fig. 5B and C). Under the same conditions, both the *S. aureus* USA300 and *S. epidermidis* SeSK1 evolved DAP-R 4 days later, at passage day 5 (Fig. 5B and C), while the *S. epidermidis* isolate SeSK1 struggled to maintain DAP-R over the complete passage, falling under the resistance cutoff every other day or showing no profound increase in daptomycin MIC (Fig. 5B and C). Thus, *S. capitis* DSM6717 and NRCS-A evolve daptomycin resistance faster than *S. epidermidis* and *S. aureus* under subinhibitory pressure with either daptomycin or vancomycin. To identify genetic changes associated with day-10 endpoints, we resequenced ScSK4 (the only DAP-S NRCS-A isolate, which at baseline did not harbor a *walK* variant; Table 1) and DSM6717 after passaging and compared variants to day 0 (Table 2). Notably, DSM6717 acquired *walK* mutations under both daptomycin and vancomycin pressure and, under daptomycin pressure, additionally acquired an *mprF* substitution (S337L) matching the same position of amino acid changes observed in NRCS-A isolates (Table 1), while ScSK4 acquired a *walK* mutation under daptomycin pressure (Table 2). Thus, DAP-R evolution in *S. capitis* is associated with the acquisition of mutations in cell envelope-associated genes.

**TABLE 2:** SNPs in *S. capitis* ScSK4 and DSM6717 at day 10 after DAP-R evolution under daptomycin pressure (DAP), vancomycin pressure (VAN), or no antibiotic pressure (No ABx), compared with day 0 (see Fig. 5)^*a*^

Strain	ScSK4		DSM6717	
Antibiotic pressure	Gene	Mutation (aa)	Gene	Mutation (aa)
DAP	*walK*	Q218E	*walK*	T12N
			*mprF*	S337L
VAN	*argR_1*	Y111F	*walK*	R288S
	*arcA*	K97E	*pycA*	Q502K
	*arcB_1*	C19S	*gmuF*	E120_A131del
	*arcD*	V347D	*acsA_2*	G129G (silent)
	*arcR*	Stop K98*		
No ABx	*agrA*	N224fs		

^*a*^ SNPs in hypothetical proteins or non-coding regions are not shown.

## DISCUSSION

This study shows a high prevalence (>90%) of daptomycin resistance in *S. capitis* NRCS-A isolates from neonatal sepsis of a local NICU collection. These observations exceed the rates of DAP-R NRCS-A isolates recently reported by Wirth et al., in which only 60% of isolates exhibited a DAP-R phenotype (14). *S. capitis* DAP-R was not attributable to a single mutation. We rather observed multiple genetic variants in genes previously linked to DAP-R in *S. aureus*. Despite variants in lipid synthesis genes such as *mprF*, *cls2*, and *pgsA,* phospholipid compositions (PG, CL, Lys-PG) of NRCS-A isolates were not altered in comparison to the DAP-S control *S. capitis* DSM6717. In contrast, in several Gram-positive pathogens, particularly viridans streptococci, mutations in *pgsA* are associated with marked depletion of PG and CL and altered daptomycin-membrane interactions, while in enterococci daptomycin resistance can involve *cls* mutations and regulatory pathways that redistribute anionic phospholipids away from the septum (60, 61). Thus, in NRCS-A, *cls2/pgsA* variants may affect membrane organization or domain localization rather than bulk phospholipid abundance (62). Invasive NRCS-A isolates displayed the common *S. aureus* DAP-R features of an increased cell surface charge and thicker cell walls compared to control *S. capitis*, but these traits did not distinguish DAP-R from DAP-S within NRCS-A. Of note, three of our isolates originated from NEC, two isolates from a same-time bloodstream isolation and intraoperative swab from a single patient with NEC and intestinal perforation. While this is compatible with intestinal involvement in this case, our data do not allow us to determine the directionality or route of infection. Nonetheless, NRCS-A has been reported to colonize the neonatal gut/rectum and gut/rectal carriage can coincide with bloodstream infection, supporting the plausibility of a gut-associated source in a subset of LOS cases (63, 64).

Our results refine how *S. capitis* differs from *S. aureus* in the evolution of DAP-R. In *S. aureus*, non-susceptibility to daptomycin frequently involves *mprF* gain-of-function and lipid remodeling (elevated Lys-PG and/or altered PG/CL distributions), accompanied by mutations in cell envelope regulators (e.g., *walKR*, *vraRS*) and transcriptional machinery (*rpoB/C*) (37). Prior experimental DAP-R evolution work in *S. capitis*/CoNS identified the two-component regulatory system *walKR* controlling cell wall biogenesis as a key factor for DAP-R in the absence of any modification of cell surface charge (40). Consistently, we found mutations in *walK* in our NRCS-A isolates which phenotypically show thickening of the cell wall. Contrary to the work of Jiang et al. (40) on other *S. capitis* isolates, we identified genetic variants in lipid synthesis genes *mprF*, *cls2*, and *pgsA* and a phenotypic increase in cell surface charge in *S. capitis* NRCS-A. Mechanistically, this dovetails with evidence that daptomycin interacts with PG and cell wall biosynthesis precursors, placing the cell envelope architecture at the center of DAP-R evolution (28, 65). The varying findings for DAP-R in CoNS underscore that rather than a single driver, multifactorial genetic and phenotypical traits may cumulate and subsequently lead to DAP-R in *S. capitis*.

Experimentally, *S. capitis* evolved DAP-R more rapidly and with greater stability than *S. aureus* and *S. epidermidis* in passaging experiments under both subinhibitory daptomycin or vancomycin pressure. Notably, although ScSK4 was the only DAP-S NRCS-A isolate and did not harbor a baseline *walK* variant, daptomycin passaging selected a *walK* mutation by day 10, underscoring *walK*/WalKR as a recurring genetic target during DAP-R evolution in *S. capitis*. In parallel, non-invasive DSM6717 acquired *walK* mutations under both daptomycin and vancomycin pressure and an *mprF* substitution under daptomycin pressure that matches the position of amino acid change observed among NRCS-A isolates. Together, these findings support that DAP-R trajectories in *S. capitis* are frequently shaped by mutations in cell envelope-associated genes. The rapid adaptive capacity of *S. capitis* echoes prior observations that NRCS-A rapidly adapts to vancomycin treatment and that cell wall thickening can diminish glycopeptide access to the bacterial cell (16, 66). Such cell envelope alterations, especially if combined with increased cell surface charge, may indirectly affect accessibility of daptomycin. Beyond cell-envelope remodeling, decreased tricarboxylic acid (TCA) cycle activity has been reported in DAP-R *S. aureus* (67), raising the possibility that metabolic rewiring may also contribute to the envelope-centric daptomycin adaptation we observe in NRCS-A. Reports of vancomycin-daptomycin cross-resistance in *S. aureus,* which seem to involve both *mprF* and *walK* (38), further support the idea that overlapping cell envelope homeostasis factors can shape resistance trajectories. The intrinsic predisposition of *S. capitis* to resistance acquisition of *S. capitis* may represent a key factor underlying the clonal success of NRCS-A in NICUs where antibiotics of last resort such as vancomycin are frequently applied. Surprisingly, only one patient received vancomycin prior to NRCS-A bloodstream infection, supporting the hypothesis of an intrinsically high predisposition to resistance and/or other factors driving resistance evolution in NRCS-A. Our findings further indicate that daptomycin efficacy may be fragile when considered for treatment of NRCS-A isolates (17) and careful vigilance for rapid MIC shifts is warranted in the NICU setting.

This work has limitations. We present a single-center strain collection and a moderate-sized set of bloodstream isolates. Owing to the limited genetic amenability of *S. capitis*, our findings lack functional validation of individual genetic variants, while our phenotypes, rather than discriminating, enable DAP-R in *S. capitis* NRCS-A. Future studies should prioritize the development of a genetic toolbox for *S. capitis* and the construction of genetic candidate variants for DAP-R.

In conclusion, we show that DAP-R in *S. capitis* NRCS-A appears to emerge via species-specific, cell-envelope-centric, polygenic adaptations. The absence of lipid remodeling alongside rapid resistance acquisition distinguishes *S. capitis* from *S. aureus* and extends prior work on NRCS-A adaptation under vancomycin, providing a mechanistic framework for future functional studies.

## SUPPLEMENTAL MATERIAL

Fig. S1 aac.01414-25-s0001.tiff Microbroth dilution assay of *S. capitis* NRCS-A isolate ScSK1 in the presence of increasing concentrations of BODIPY-labeled daptomycin, as indicated.

Fig. S2 aac.01414-25-s0002.tiff Representative TLC image of extracted lipids (PG, CL, Lys-PG, as indicated) from *S. aureus* WT (SA WT), S. aureus ΔmprF (SA ΔmprF), and *S. capitis* (DSM6717, ScSK1-6).

Supplemental material aac.01414-25-s0003.docx Tables S1 and S2; Fig. S1 and S2 captions.

ASM does not own the copyrights to Supplemental Material that may be linked to, or accessed through, an article. The authors have granted ASM a non-exclusive, world-wide license to publish the Supplemental Material files. Please contact the corresponding author directly for reuse.

## References

[1] Murray CJL, Ikuta KS, Sharara F, Swetschinski L, Robles Aguilar G, Gray A, Han C, Bisignano C, Rao P, Wool E, et al.. 2022. Global burden of bacterial antimicrobial resistance in 2019: a systematic analysis. Lancet 399:629–655. doi:10.1016/S0140-6736(21)02724-0 35065702 PMC8841637

[2] Wattal C, Kler N, Oberoi JK, Fursule A, Kumar A, Thakur A. 2020. Neonatal sepsis: mortality and morbidity in neonatal sepsis due to multidrug-resistant (MDR) organisms: part 1. Indian J Pediatr 87:117–121. doi:10.1007/s12098-019-03106-z 31828600 PMC6974503

[3] Tzialla C, Borghesi A, Pozzi M, Stronati M. 2015. Neonatal infections due to multi-resistant strains: epidemiology, current treatment, emerging therapeutic approaches and prevention. Clin Chim Acta 451:71–77. doi:10.1016/j.cca.2015.02.038 25749408

[4] Zou H, Jia X, He X, Su Y, Zhou L, Shen Y, Sheng C, Liao A, Li C, Li Q. 2021. Emerging threat of multidrug resistant pathogens from neonatal sepsis. Front Cell Infect Microbiol 11:694093. doi:10.3389/fcimb.2021.694093 34322398 PMC8312093

[5] Yusef D, Shalakhti T, Awad S, Algharaibeh H, Khasawneh W. 2018. Clinical characteristics and epidemiology of sepsis in the neonatal intensive care unit in the era of multi-drug resistant organisms: a retrospective review. Pediatr Neonatol 59:35–41. doi:10.1016/j.pedneo.2017.06.001 28642139

[6] Dong Y, Speer CP. 2015. Late-onset neonatal sepsis: recent developments. Arch Dis Child Fetal Neonatal Ed 100:F257–63. doi:10.1136/archdischild-2014-306213 25425653 PMC4413803

[7] Köstlin-Gille N, Härtel C, Haug C, Göpel W, Zemlin M, Müller A, Poets CF, Herting E, Gille C. 2021. Epidemiology of early and late onset neonatal sepsis in very low birthweight infants: data from the german neonatal network. Pediatr Infect Dis J 40:255–259. doi:10.1097/INF.0000000000002976 33538544

[8] Fleischmann C, Reichert F, Cassini A, Horner R, Harder T, Markwart R, Tröndle M, Savova Y, Kissoon N, Schlattmann P, Reinhart K, Allegranzi B, Eckmanns T. 2021. Global incidence and mortality of neonatal sepsis: a systematic review and meta-analysis. Arch Dis Child 106:745–752. doi:10.1136/archdischild-2020-320217 33483376 PMC8311109

[9] Niño DF, Sodhi CP, Hackam DJ. 2016. Necrotizing enterocolitis: new insights into pathogenesis and mechanisms. Nat Rev Gastroenterol Hepatol 13:590–600. doi:10.1038/nrgastro.2016.119 27534694 PMC5124124

[10] Bizzarro MJ, Raskind C, Baltimore RS, Gallagher PG. 2005. Seventy-five years of neonatal sepsis at Yale: 1928–2003. Pediatrics 116:595–602. doi:10.1542/peds.2005-0552 16140698

[11] Flannery DD, Edwards EM, Coggins SA, Horbar JD, Puopolo KM. 2022. Late-onset sepsis among very preterm infants. Pediatrics 150:e2022058813. doi:10.1542/peds.2022-058813 36366916 PMC11151779

[12] Rasigade JP, Raulin O, Picaud JC, Tellini C, Bes M, Grando J, Ben Saïd M, Claris O, Etienne J, Tigaud S, Laurent F. 2012. Methicillin-resistant Staphylococcus capitis with reduced vancomycin susceptibility causes late-onset sepsis in intensive care neonates. PLoS One 7:e31548. doi:10.1371/journal.pone.0031548 22348102 PMC3279402

[13] Simões PM, Lemriss H, Dumont Y, Lemriss S, Rasigade J-P, Assant-Trouillet S, Ibrahimi A, El Kabbaj S, Butin M, Laurent F. 2016. Single-molecule sequencing (PacBio) of the Staphylococcus capitis NRCS-A clone reveals the basis of multidrug resistance and adaptation to the neonatal intensive care unit environment. Front Microbiol 7:1991. doi:10.3389/fmicb.2016.01991 28018320 PMC5157051

[14] Wirth T, Bergot M, Rasigade J-P, Pichon B, Barbier M, Martins-Simoes P, Jacob L, Pike R, Tissieres P, Picaud J-C, Kearns A, Supply P, Butin M, Laurent F, International Consortium for Staphylococcus capitis neonatal sepsis, ESGS Study Group of ESCMID. 2020. Niche specialization and spread of Staphylococcus capitis involved in neonatal sepsis. Nat Microbiol 5:735–745. doi:10.1038/s41564-020-0676-2 32341568

[15] Butin M, Rasigade J-P, Martins-Simões P, Meugnier H, Lemriss H, Goering RV, Kearns A, Deighton MA, Denis O, Ibrahimi A, Claris O, Vandenesch F, Picaud J-C, Laurent F. 2016. Wide geographical dissemination of the multiresistant Staphylococcus capitis NRCS-A clone in neonatal intensive-care units. Clin Microbiol Infect 22:46–52. doi:10.1016/j.cmi.2015.09.008 26404028

[16] Butin M, Martins-Simões P, Picaud JC, Kearns A, Claris O, Vandenesch F, Laurent F, Rasigade JP. 2015. Adaptation to vancomycin pressure of multiresistant Staphylococcus capitis NRCS-A involved in neonatal sepsis. J Antimicrob Chemother 70:3027–3031. doi:10.1093/jac/dkv217 26203181

[17] Papachatzi E, Gkentzi D, Tzifas S, Dassios T, Dimitriou G. 2024. Daptomycin use for persistent coagulase-negative staphylococcal bacteremia in a neonatal intensive care unit. Antibiotics (Basel) 13:254. doi:10.3390/antibiotics13030254 38534689 PMC10967625

[18] Mohzari Y, Aljobair F, Alrashed A, Asdaq SMB, Alshuraim RA, Asfour SS, Al-Mouqdad MM, Bamogaddam RF, Al-Anazi D, Zeilinger CE, Alamer A, Alhassan BM, Sreeharsha N. 2021. Safety and efficacy of daptomycin in neonates with coagulase-negative staphylococci: case series analysis. Antibiotics (Basel) 10:168. doi:10.3390/antibiotics10020168 33562197 PMC7915314

[19] Coggins SA, Glaser K. 2022. Updates in late-onset sepsis: risk assessment, therapy, and outcomes. Neoreviews 23:738–755. doi:10.1542/neo.23-10-e738 36316254 PMC9675597

[20] Eliopoulos GM, Willey S, Reiszner E, Spitzer PG, Caputo G, Moellering RC Jr. 1986. In vitro and in vivo activity of LY 146032, a new cyclic lipopeptide antibiotic. Antimicrob Agents Chemother 30:532–535. doi:10.1128/AAC.30.4.532 3024560 PMC176475

[21] Jones RN, Barry AL. 1987. Antimicrobial activity and spectrum of LY146032, a lipopeptide antibiotic, including susceptibility testing recommendations. Antimicrob Agents Chemother 31:625–629. doi:10.1128/AAC.31.4.625 3038001 PMC174792

[22] Lakey JH, Ptak M. 1988. Fluorescence indicates a calcium-dependent interaction between the lipopeptide antibiotic LY146032 and phospholipid membranes. Biochemistry 27:4639–4645. doi:10.1021/bi00413a009 2844233

[23] Fiore M, Alfieri A, Fiore D, Iuliano P, Spatola FG, Limone A, Pezone I, Leone S. 2025. Use of daptomycin to manage severe MRSA infections in humans. Antibiotics (Basel) 14:617. doi:10.3390/antibiotics14060617 40558207 PMC12190081

[24] Seaton RA, Gonzalez-Ruiz A, Cleveland KO, Couch KA, Pathan R, Hamed K. 2016. Real-world daptomycin use across wide geographical regions: results from a pooled analysis of CORE and EU-CORE. Ann Clin Microbiol Antimicrob 15:18. doi:10.1186/s12941-016-0130-8 26976128 PMC4791778

[25] Rimal B, Chang J, Liu C, Rashid R, Singh M, Kim SJ. 2023. The effects of daptomycin on cell wall biosynthesis in Enterococcal faecalis. Sci Rep 13:12227. doi:10.1038/s41598-023-39486-8 37507537 PMC10382475

[26] Kotsogianni I, Wood TM, Alexander FM, Cochrane SA, Martin NI. 2021. Binding studies reveal phospholipid specificity and its role in the calcium-dependent mechanism of action of daptomycin. ACS Infect Dis 7:2612–2619. doi:10.1021/acsinfecdis.1c00316 34406007 PMC8438661

[27] Machhua P, Unnithan VG, Liu Y, Jiang Y, Zhang L, Guo Z. 2025. Daptomycin forms a stable complex with phosphatidylglycerol for selective uptake to bacterial membrane. eLife 13:RP93267. doi:10.7554/eLife.93267 40455071 PMC12129450

[28] Grein F, Müller A, Scherer KM, Liu X, Ludwig KC, Klöckner A, Strach M, Sahl H-G, Kubitscheck U, Schneider T. 2020. Ca^2+^-Daptomycin targets cell wall biosynthesis by forming a tripartite complex with undecaprenyl-coupled intermediates and membrane lipids. Nat Commun 11:1455. doi:10.1038/s41467-020-15257-1 32193379 PMC7081307

[29] Zhang T, Muraih JK, MacCormick B, Silverman J, Palmer M. 2014. Daptomycin forms cation- and size-selective pores in model membranes. Biochim Biophys Acta 1838:2425–2430. doi:10.1016/j.bbamem.2014.05.014 24857935

[30] Boudjemaa R, Cabriel C, Dubois-Brissonnet F, Bourg N, Dupuis G, Gruss A, Lévêque-Fort S, Briandet R, Fontaine-Aupart MP, Steenkeste K. 2018. Impact of bacterial membrane fatty acid composition on the failure of daptomycin to kill Staphylococcus aureus. Antimicrob Agents Chemother 62:e00023-18. doi:10.1128/AAC.00023-18 29735564 PMC6021656

[31] Yin Y, Chen H, Li S, Gao H, Sun S, Li H, Wang R, Jin L, Liu Y, Wang H. 2019. Daptomycin resistance in methicillin-resistant Staphylococcus aureus is conferred by IS256 insertion in the promoter of mprF along with mutations in mprF and walK. Int J Antimicrob Agents 54:673–680. doi:10.1016/j.ijantimicag.2019.08.021 31479743

[32] Sabat AJ, Tinelli M, Grundmann H, Akkerboom V, Monaco M, Del Grosso M, Errico G, Pantosti A, Friedrich AW. 2018. Daptomycin resistant Staphylococcus aureus clinical strain with novel non-synonymous mutations in the mprF and vraS genes: a new insight into daptomycin resistance. Front Microbiol 9:2705. doi:10.3389/fmicb.2018.02705 30459746 PMC6232378

[33] Bayer AS, Schneider T, Sahl HG. 2013. Mechanisms of daptomycin resistance in Staphylococcus aureus: role of the cell membrane and cell wall. Ann N Y Acad Sci 1277:139–158. doi:10.1111/j.1749-6632.2012.06819.x 23215859 PMC3556211

[34] Xu Y, Xiao Y, Zhao H, Wang B, Yu J, Shang Y, Zhou Y, Wu X, Guo Y, Yu F. 2024. Phenotypic and genetic characterization of daptomycin non-susceptible Staphylococcus aureus strains selected by adaptive laboratory evolution. Front Cell Infect Microbiol 14:1453233. doi:10.3389/fcimb.2024.1453233 39512591 PMC11540788

[35] Ma Z, Lasek-Nesselquist E, Lu J, Schneider R, Shah R, Oliva G, Pata J, McDonough K, Pai MP, Rose WE, Sakoulas G, Malik M. 2018. Characterization of genetic changes associated with daptomycin nonsusceptibility in Staphylococcus aureus. PLoS One 13:e0198366. doi:10.1371/journal.pone.0198366 29879195 PMC5991675

[36] Freeman CD, Hansen T, Urbauer R, Wilkinson BJ, Singh VK, Hines KM. 2024. Defective pgsA contributes to increased membrane fluidity and cell wall thickening in S. aureus with high-level daptomycin resistance. mSphere 9:e0011524. doi:10.1101/2023.04.11.536441 38752757 PMC11332330

[37] Jiang S, Chen M, Zhang J, Ba X, Zhang H, Hong Y, Sun L, Wang Z, Zhuang H, Zhu F, Chen Y, Wang H, Zhao F, Chen Y, Yu Y, Ji S. 2023. Profiling daptomycin resistance among diverse methicillin-resistant Staphylococcus aureus lineages in China. Antimicrob Agents Chemother 67:e0056323. doi:10.1128/aac.00563-23 37902403 PMC10649010

[38] Chen CJ, Huang YC, Chiu CH. 2015. Multiple pathways of cross-resistance to glycopeptides and daptomycin in persistent MRSA bacteraemia. J Antimicrob Chemother 70:2965–2972. doi:10.1093/jac/dkv225 26216581

[39] Ernst CM, Peschel A. 2011. Broad-spectrum antimicrobial peptide resistance by MprF-mediated aminoacylation and flipping of phospholipids. Mol Microbiol 80:290–299. doi:10.1111/j.1365-2958.2011.07576.x 21306448

[40] Jiang JH, Dexter C, Cameron DR, Monk IR, Baines SL, Abbott IJ, Spelman DW, Kostoulias X, Nethercott C, Howden BP, Peleg AY. 2019. Evolution of daptomycin resistance in coagulase-negative staphylococci involves mutations of the essential two-component regulator WalKR. Antimicrob Agents Chemother 63:e01926-18. doi:10.1128/AAC.01926-18 30617095 PMC6395924

[41] Peschel A, Jack RW, Otto M, Collins LV, Staubitz P, Nicholson G, Kalbacher H, Nieuwenhuizen WF, Jung G, Tarkowski A, van Kessel KP, van Strijp JA. 2001. Staphylococcus aureus resistance to human defensins and evasion of neutrophil killing via the novel virulence factor MprF is based on modification of membrane lipids with l-lysine. J Exp Med 193:1067–1076. doi:10.1084/jem.193.9.1067 11342591 PMC2193429

[42] Ledger EVK, Mesnage S, Edwards AM. 2022. Human serum triggers antibiotic tolerance in Staphylococcus aureus. Nat Commun 13:2041. doi:10.1038/s41467-022-29717-3 35440121 PMC9018823

[43] Pader V, Hakim S, Painter KL, Wigneshweraraj S, Clarke TB, Edwards AM. 2016. Staphylococcus aureus inactivates daptomycin by releasing membrane phospholipids. Nat Microbiol 2:16194. doi:10.1038/nmicrobiol.2016.194 27775684

[44] Slavetinsky CJ, Hauser JN, Gekeler C, Slavetinsky J, Geyer A, Kraus A, Heilingbrunner D, Wagner S, Tesar M, Krismer B, Kuhn S, Ernst CM, Peschel A. 2022. Sensitizing Staphylococcus aureus to antibacterial agents by decoding and blocking the lipid flippase MprF. eLife 11:e66376. doi:10.7554/eLife.66376 35044295 PMC8806190

[45] Beck C, Krusche J, Notaro A, Walter A, Kränkel L, Vollert A, Stemmler R, Wittmann J, Schaller M, Slavetinsky C, Mayer C, De Castro C, Peschel A. 2024. Wall teichoic acid substitution with glucose governs phage susceptibility of Staphylococcus epidermidis. mBio 15:e0199023. doi:10.1128/mbio.01990-23 38470054 PMC11005348

[46] BLIGH EG, DYER WJ. 1959. A rapid method of total lipid extraction and purification. Can J Biochem Physiol 37:911–917. doi:10.1139/o59-099 13671378

[47] Ernst CM, Staubitz P, Mishra NN, Yang SJ, Hornig G, Kalbacher H, Bayer AS, Kraus D, Peschel A. 2009. The bacterial defensin resistance protein MprF consists of separable domains for lipid lysinylation and antimicrobial peptide repulsion. PLoS Pathog 5:e1000660. doi:10.1371/journal.ppat.1000660 19915718 PMC2774229

[48] Winstel V, Liang C, Sanchez-Carballo P, Steglich M, Munar M, Bröker BM, Penadés JR, Nübel U, Holst O, Dandekar T, Peschel A, Xia G. 2013. Wall teichoic acid structure governs horizontal gene transfer between major bacterial pathogens. Nat Commun 4:2345. doi:10.1038/ncomms3345 23965785 PMC3903184

[49] Peschel A, Otto M, Jack RW, Kalbacher H, Jung G, Götz F. 1999. Inactivation of the dlt operon in Staphylococcus aureus confers sensitivity to defensins, protegrins, and other antimicrobial peptides. J Biol Chem 274:8405–8410. doi:10.1074/jbc.274.13.8405 10085071

[50] Ernst CM, Slavetinsky CJ, Kuhn S, Hauser JN, Nega M, Mishra NN, Gekeler C, Bayer AS, Peschel A. 2018. Gain-of-function mutations in the phospholipid flippase MprF confer specific daptomycin resistance. mBio 9:e01659-18. doi:10.1128/mBio.01659-18 30563904 PMC6299216

[51] Friedman L, Alder JD, Silverman JA. 2006. Genetic changes that correlate with reduced susceptibility to daptomycin in Staphylococcus aureus. Antimicrob Agents Chemother 50:2137–2145. doi:10.1128/AAC.00039-06 16723576 PMC1479123

[52] Peleg AY, Miyakis S, Ward DV, Earl AM, Rubio A, Cameron DR, Pillai S, Moellering RC, Eliopoulos GM. 2012. Whole genome characterization of the mechanisms of daptomycin resistance in clinical and laboratory derived isolates of Staphylococcus aureus. PLoS One 7:e28316. doi:10.1371/journal.pone.0028316 22238576 PMC3253072

[53] Fischer A, Yang S-J, Bayer AS, Vaezzadeh AR, Herzig S, Stenz L, Girard M, Sakoulas G, Scherl A, Yeaman MR, Proctor RA, Schrenzel J, François P. 2011. Daptomycin resistance mechanisms in clinically derived Staphylococcus aureus strains assessed by a combined transcriptomics and proteomics approach. J Antimicrob Chemother 66:1696–1711. doi:10.1093/jac/dkr195 21622973 PMC3133485

[54] Mishra NN, Bayer AS. 2013. Correlation of cell membrane lipid profiles with daptomycin resistance in methicillin-resistant Staphylococcus aureus*.* Antimicrob Agents Chemother 57:1082–1085. doi:10.1128/AAC.02182-12 23254419 PMC3553710

[55] Nguyen AH, Hood KS, Mileykovskaya E, Miller WR, Tran TT. 2022. Bacterial cell membranes and their role in daptomycin resistance: a review. Front Mol Biosci 9:1035574. doi:10.3389/fmolb.2022.1035574 36452455 PMC9702088

[56] Jiang JH, Bhuiyan MS, Shen HH, Cameron DR, Rupasinghe TWT, Wu CM, Le Brun AP, Kostoulias X, Domene C, Fulcher AJ, McConville MJ, Howden BP, Lieschke GJ, Peleg AY. 2019. Antibiotic resistance and host immune evasion in Staphylococcus aureus mediated by a metabolic adaptation. Proc Natl Acad Sci USA 116:3722–3727. doi:10.1073/pnas.1812066116 30808758 PMC6397524

[57] Howden BP, McEvoy CRE, Allen DL, Chua K, Gao W, Harrison PF, Bell J, Coombs G, Bennett-Wood V, Porter JL, Robins-Browne R, Davies JK, Seemann T, Stinear TP. 2011. Evolution of multidrug resistance during Staphylococcus aureus infection involves mutation of the essential two component regulator WalKR. PLoS Pathog 7:e1002359. doi:10.1371/journal.ppat.1002359 22102812 PMC3213104

[58] Kuroda M, Kuroda H, Oshima T, Takeuchi F, Mori H, Hiramatsu K. 2003. Two-component system VraSR positively modulates the regulation of cell-wall biosynthesis pathway in Staphylococcus aureus. Mol Microbiol 49:807–821. doi:10.1046/j.1365-2958.2003.03599.x 12864861

[59] Sharif S, Singh M, Kim SJ, Schaefer J. 2009. Staphylococcus aureus peptidoglycan tertiary structure from carbon-13 spin diffusion. J Am Chem Soc 131:7023–7030. doi:10.1021/ja808971c 19419167 PMC2778264

[60] Nguyen AH, Tran TT, Panesso D, Hood KS, Polamraju V, Zhang R, Khan A, Miller WR, Mileykovskaya E, Shamoo Y, Xu L, Vitrac H, Arias CA. 2024. Molecular basis of cell membrane adaptation in daptomycin-resistant Enterococcus faecalis. JCI Insight 9:e173836. doi:10.1172/jci.insight.173836 39405116 PMC11601895

[61] Tran TT, Mishra NN, Seepersaud R, Diaz L, Rios R, Dinh AQ, Garcia-de-la-Maria C, Rybak MJ, Miro JM, Shelburne SA, Sullam PM, Bayer AS, Arias CA. 2019. Mutations in cdsA and pgsA correlate with daptomycin resistance in Streptococcus mitis and S. oralis. Antimicrob Agents Chemother 63. doi:10.1128/AAC.01531-18 PMC6355606 30509945

[62] Hines KM, Waalkes A, Penewit K, Holmes EA, Salipante SJ, Werth BJ, Xu L. 2017. Characterization of the mechanisms of daptomycin resistance among gram-positive bacterial pathogens by multidimensional lipidomics. mSphere 2:e00492-17. doi:10.1128/mSphere.00492-17 29242835 PMC5729219

[63] Felgate H, Sethi D, Faust K, Kiy C, Härtel C, Rupp J, Clifford R, Dean R, Tremlett C, Wain J, Langridge G, Clarke P, Page AJ, Webber MA. 2023. Characterisation of neonatal Staphylococcus capitis NRCS-A isolates compared with non NRCS-A Staphylococcus capitis from neonates and adults. Microb Genom 9:001106. doi:10.1099/mgen.0.001106 37791541 PMC10634448

[64] Lees EA, Gentry J, Webster H, Sanderson N, Eyre D, Wilson D, Lipworth S, Crook D, Wong THN, Mark A, Jeffery K, Paulus S, Young BC. 2025. Multiple introductions of NRCS-A Staphylococcus capitis to the neonatal intensive care unit drive neonatal bloodstream infections: a case-control and environmental genomic survey. Microb Genom 11:001340. doi:10.1099/mgen.0.001340 39773387 PMC11706212

[65] Miller WR, Bayer AS, Arias CA. 2016. Mechanism of action and resistance to daptomycin in Staphylococcus aureus and enterococci. Cold Spring Harb Perspect Med 6:a026997. doi:10.1101/cshperspect.a026997 27580748 PMC5088507

[66] Cui L, Ma X, Sato K, Okuma K, Tenover FC, Mamizuka EM, Gemmell CG, Kim MN, Ploy MC, El-Solh N, Ferraz V, Hiramatsu K. 2003. Cell wall thickening is a common feature of vancomycin resistance in Staphylococcus aureus. J Clin Microbiol 41:5–14. doi:10.1128/JCM.41.1.5-14.2003 12517819 PMC149586

[67] Gaupp R, Lei S, Reed JM, Peisker H, Boyle-Vavra S, Bayer AS, Bischoff M, Herrmann M, Daum RS, Powers R, Somerville GA. 2015. Staphylococcus aureus metabolic adaptations during the transition from a daptomycin susceptibility phenotype to a daptomycin nonsusceptibility phenotype. Antimicrob Agents Chemother 59:4226–4238. doi:10.1128/AAC.00160-15 25963986 PMC4468685

[68] Lemriss H, Martins Simoes P, Lemriss S, Butin M, Ibrahimi A, El Kabbaj S, Rasigade J, Laurent F. 2014. Non-contiguous finished genome sequence of Staphylococcus capitis CR01 (pulsetype NRCS-A). Stand Genomic Sci 9:1118–1127. doi:10.4056/sigs.5491045 25197487 PMC4149024

