# Supplementary figures and images for: Polygenic, cell-envelope adaptations drive high-frequency daptomycin resistance in Staphylococcus capitis NRCS-A from neonatal sepsis and NEC
Source: Antimicrob Agents Chemother. 2026 Mar 24;70(5):e01414-25. doi: 10.1128/aac.01414-25 (PMC13148029; doi:10.1128/aac.01414-25)

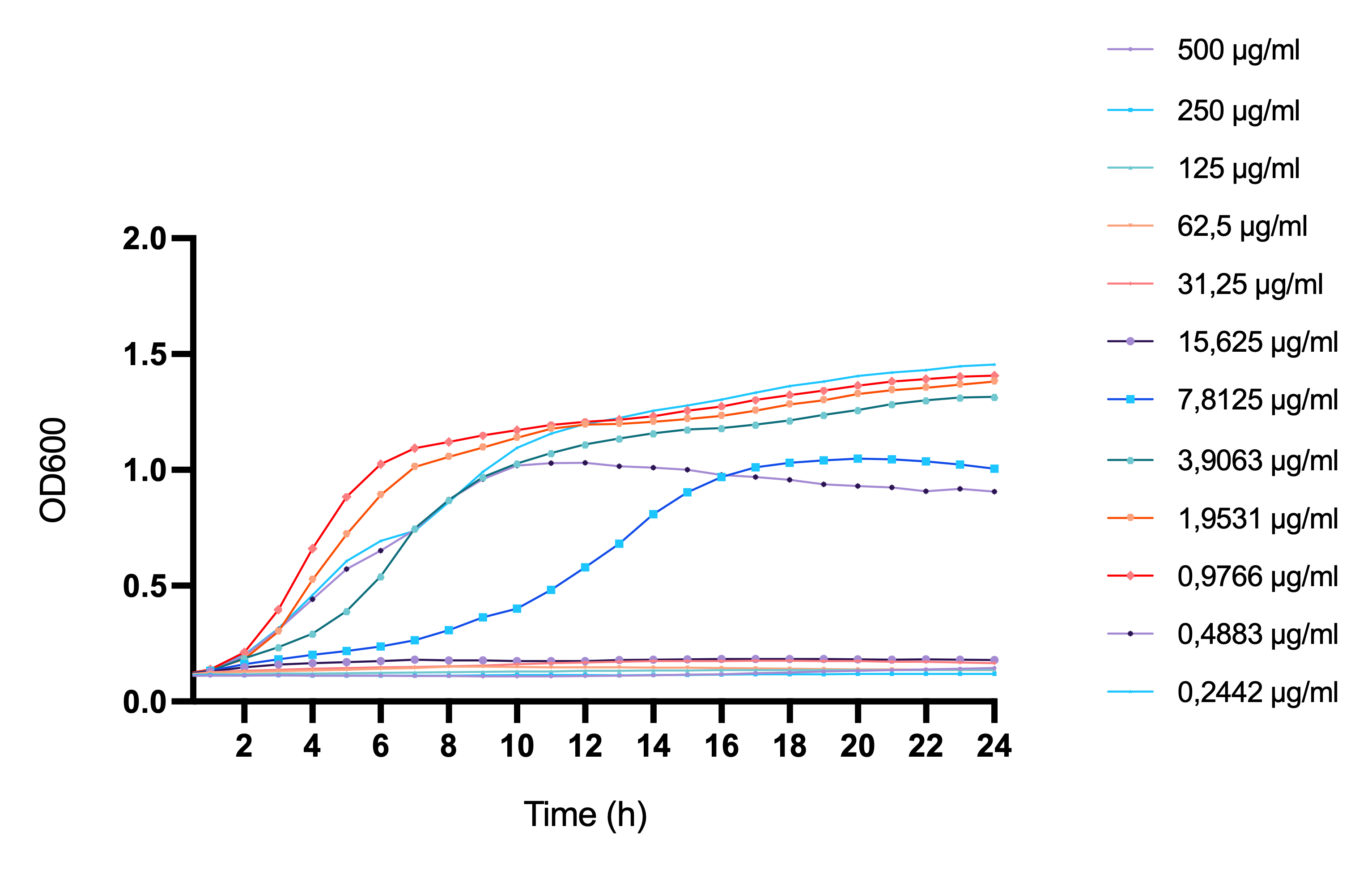

Supplement: Fig. S1 — Microbroth dilution assay of S. capitis NRCS-A isolate ScSK1 in the presence of increasing concentrations of BODIPY-labeled daptomycin, as indicated. [file aac.01414-25-s0001.tiff]

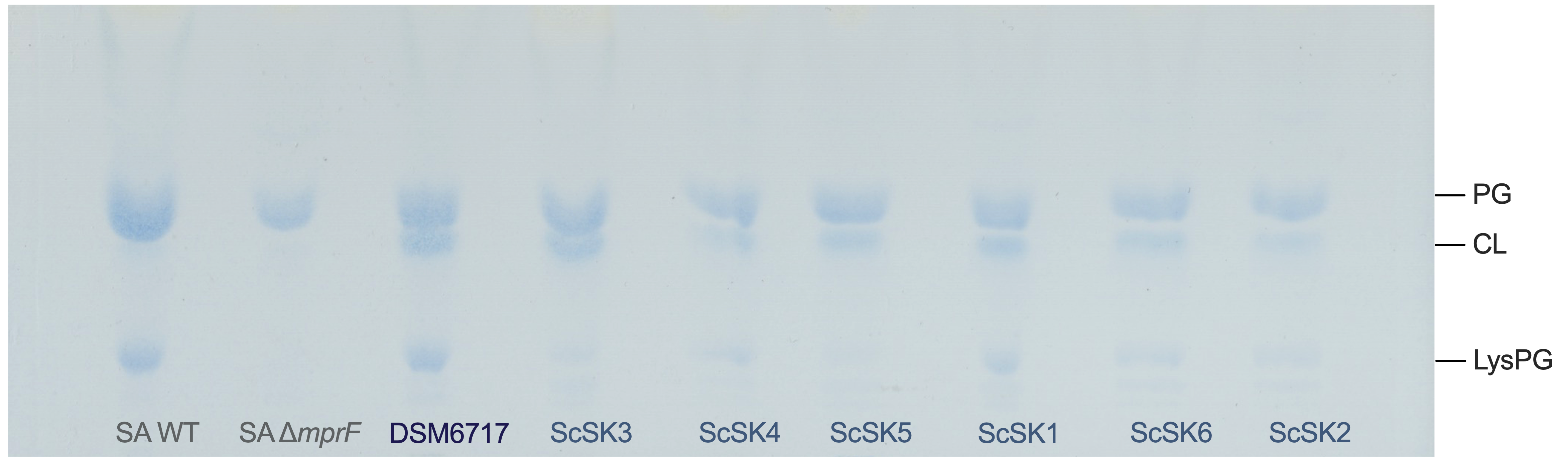

Supplement: Fig. S2 — Representative TLC image of extracted lipids (PG, CL, Lys-PG, as indicated) from S. aureus WT (SA WT), S. aureus ΔmprF (SA ΔmprF), and S. capitis (DSM6717, ScSK1-6). [file aac.01414-25-s0002.tiff]
