# Supplementary material for: Polygenic, cell-envelope adaptations drive high-frequency daptomycin resistance in Staphylococcus capitis NRCS-A from neonatal sepsis and NEC
Source: Antimicrob Agents Chemother. 2026 Mar 24;70(5):e01414-25. doi: 10.1128/aac.01414-25 (PMC13148029; doi:10.1128/aac.01414-25)
Supplement: Supplemental material — Tables S1 and S2; Fig. S1 and S2 captions. [file aac.01414-25-s0003.docx]

**Supplementary material**

Supplementary tables

**Table S1**: Bacterial strain collection. Abbreviations: Gentamicin (GEN), oxacillin (OX), erythromycin (ERY), clindamycin (CD), fosfomycin (FOS), and University Hospital Tübingen (UHT)

| **Species** | **Strain** | **Origin** | **Other antibiotic resistances** |
| --- | --- | --- | --- |
| *S. capitis* | ScSK1 | Bloodstream isolate from neonatal sepsis, UHT | GEN, OX, ERY, CD, FOS |
| *S. capitis* | ScSK2 | Bloodstream isolate from NEC with neonatal sepsis, UHT | GEN, OX, ERY, CD, FOS |
| *S. capitis* | ScSK3 | Bloodstream isolate from neonatal sepsis, UHT | GEN, OX, ERY, CD, FOS |
| *S. capitis* | ScSK4 | Bloodstream isolate from neonatal sepsis, UHT | GEN, OX, ERY, CD, FOS |
| *S. capitis* | ScSK5 | Bloodstream isolate from neonatal sepsis, UHT | GEN, OX, FOS |
| *S. capitis* | ScSK6 | Bloodstream isolate from neonatal sepsis, UHT | GEN, OX, FOS |
| *S. capitis* | ScSK7 | Bloodstream isolate from neonatal sepsis, UHT | GEN, OX, ERY, CD, FOS |
| *S. capitis* | ScSK8 | Bloodstream isolate from neonatal sepsis, UHT | GEN, OX, FOS |
| *S. capitis* | ScSK9 | Intraoperative swab (stool in the abdominal cavity) from NEC with neonatal sepsis (corresponding to ScKS10) | GEN, OX, ERY, CD, FOS |
| *S. capitis* | ScSK10 | Bloodstream isolate from NEC with neonatal sepsis (corresponding to ScKS9) | GEN, OX, ERY, CD, FOS |
| *S. capitis* | ScSK11 | Bloodstream isolate from sepsis, UHT; This is no isolate from neonatal sepsis, but from a catheter sepsis from a toddler. | n/d |
| *S. capitis* | DSM6717 | Human skin isolate from the German Collection of Microorganisms and Cell Cultures (DSMZ). | n/d |
| *S. epidermidis* | SeSK1 | Clinical bloodstream isolate, UHT | n/d |
| *S. aureus* | USA300 | McDougal et al. 2003 | n/d |
| *S. aureus* | SA113 | Iordanescu and Surdeanu, 1976 | n/d |
| *S. aureus* | SA113 ∆*mprf* | Peschel et al., 2001 | n/d |

**Table S2:** Antibiotic exposure of *S. capitis* NRCS-A strains prior to the bloodstream infection,

order of antibiotics according to time of admission

| **Strain** | **Antibiotic exposure prior to bloodstream infection**  **(duration in days)** |
| --- | --- |
| ScSK1 | Ampicillin (5), Tobramycin (5) |
| ScSK2 | Ampicillin (2), Tobramycin (2), Amikacin (7), Imipinem (7) |
| ScSK3 | Ampicillin (10), Tobramycin (4), Amikacin (8), Cefotaxim (5), Imipinem (4) |
| ScSK4 | Ampicillin (5), Tobramycin (5), Amikacin (4), Imipinem (7) |
| ScSK5 | Ampicillin (2), Tobramycin (2) |
| ScSK6 | Imipinem (8), Vancomycin (7), Amikacin (3), Ampicillin (3), Cefotaxim (3) |
| ScSK7 | Ampicillin (28), Tobramycin (23) |
| ScSK8 | Ampicillin (5), Tobramycin (5), Cefotaxim (5), Amikacin (5), Meropenem (5) |
| ScSK9 | Ampicillin (2), Tobramycin (2) |
| ScSK10 | Ampicillin (2), Tobramycin (2) |
| ScSK11 | Piperacillin (2), Tazobactam (2), Cotrimoxazol (1) |

Supplementary figure legends

**Figure S1.** Microbroth dilution assay of *S. capitis* NRCS-A isolate ScSK1 in the presence of increasing concentrations of BODIPY-labeled daptomycin, as indicated.

**Figure S2.** Representative TLC image of extracted lipids (PG, CL, Lys-PG, as indicated) from S. aureus WT (SA WT), S. aureus ΔmprF (SA ΔmprF), and S. capitis (DSM6717, ScSK1–6).
